# The transmission routes of African swine fever during an outbreak in Serbia July–August 2023: African swine fever virus detections in environmental samples and insects

**DOI:** 10.3389/fvets.2024.1467273

**Published:** 2024-11-15

**Authors:** Ana Vasić, Bojan Milovanović, Dimitrije Glišić, Mihaela Kavran, Jasna Kureljušić, Aleksandar Živulj, Branislav Kureljušić, Vesna Milićević

**Affiliations:** ^1^Scientific Institute of Veterinary Medicine of Serbia, Belgrade, Serbia; ^2^Faculty of Agriculture, Centre for Excellence One Health, University of Novi Sad, Novi Sad, Serbia; ^3^Veterinarski specijalistički institut “Pančevo”, Pančevo, Serbia

**Keywords:** African swine fever virus, flies, environment, *Lucilia sericata*, *Stomoxys calcitrans*, Serbia

## Abstract

African swine fever (ASF) is a highly contagious viral disease of domestic pigs and wild boar (*Sus scrofa*) caused by an arbovirus- African swine fever virus (ASFV), which is classified into the family *Asfarviridae*. Even though the main transmission route of ASFV is direct contact between animals and carcasses releasing ASFV into the environment, the role of other transmission routes such as via environmental contamination or insects remains in great part unclear. During an epidemic f ASF in Serbia in 2023, environmental samples (soil, feed, water and swabs from the pig barns), and insects [resulting in collection of adult and/or larval stages of non-biting flies (*Diptera*: *Calliphoridae* and *Muscidae*)] were collected in four locations in South Banat district of Serbia. To assess the possibility that insects carry the ASFV in infected courtyards, sticky fly traps and open Petri dishes containing meat mixed with humid cotton wads were offered in three locations during the five days of the experiment in the Belgrade area. Furthermore, to confirm the role of flies in ASF in mechanical transmission pathway in Serbia, L2 and L3 larvae of flies (*Lucilia sericata* Meigan, 1826; *Stomoxys calcitrans* Linnaeus, 1758) were collected from the pig carcasses from infected farms, bred to adults in the Laboratory and an ASFV spiked meat meal was placed into cages with three-day-old flies (*n* = 52) to estimate positivity of flies and duration of mechanical transmission of ASFV. The results from the environmental samples showed no positive ASFV DNA detection, the same was true for the samples from meat-based traps and sticky fly traps, while ASFV DNA was detected in three samples containing eggs, L1 and L3 fly larvae collected from carcasses and adult flies (*L. sericata*). In experimental conditions, only one *S. calcitrans* fly tested positive on day 1 post-infection. The results implicate the possible role of *Lucilia* sp. flies in the mechanical transmission of ASFV as well as *S. calcitrans* in Serbia during an outbreak, while ASFV DNA was not detected in environmental samples in this study.

## Introduction

African swine fever (ASF) is a highly contagious hemorrhagic disease of domestic pigs and wild boar (*Sus scrofa*) caused by an arbovirus classified into the family *Asfarviridae* and genus *Asfivirus*. In Europe, the presence of African swine fever virus (ASFV) genotype II was first detected in 2007 in Georgia (1), and in the following years it spread in the direction of Western Europe reaching Serbia in 2019 (2). In later years ASFV has been detected in Serbia occurring in both domestic pigs and wild boar (3). Furthermore, multiple ASFV strains of genotype II have been circulating in Serbia (4). During 2022 ASFV was detected in different locations, in western Serbia (5) in domestic pigs enhanced by specific structure of swine holdings in rural areas with low biosecurity measures (6).

According to Serbian pig holding categorization given by the Veterinary Directorate, Ministry of Agriculture, there are five production types: commercial farms (high biosecurity); family farms type A (over 10 animals, high biosecurity); family farms type B (over 10 animals, low biosecurity); backyards (few pigs for self-consumption, minimal biosecurity); and free-range systems with no biosecurity. Backyard production in Serbia focuses on home consumption and local sales for extra income, often involving home slaughtering, swill feeding, and little biosecurity (7). Furthermore, this widespread type of pig production in villages in Serbia mostly does not meet even the minimum biosecurity standards. In a study, it was shown that backyard pig production is usually consisting of up to five pigs per holding, aimed at production of fatteners (59.5%), located within a hunting area or within 1,000 meters of a hunting ground (61.8%), and approximately 60.7% of holdings were situated close to other pig holdings within 100 meters distance with contact between different animal species (such as dogs, cats) in 76.4% of holdings and with. Complete fencing in 57.3% of holdings (8).

In the natural cycle in Africa where ASFV originated, soft ticks *(Acari: Argasidae)* of the genus *Ornithodoros* are reservoirs and vectors, through the population of several species of wild pigs [such as the common warthog (*Phacochoerus africanus*) and bushpigs (*Potamochoerus larvatus*)] (9). The main transmission routes of ASFV in Europe include wild boar population and human activities (10) since established population of the only competent tick vector species present in Europe, *Carios erraticus* (Lucas, 1849), formerly *Ornithodoros erraticus,* is found only in restricted areas of Iberian Peninsula (11). Possibilities of ASFV transmission via different insect species as mechanical vectors are well established for several families such as flies (*Diptera: Muscidae*) (12, 13), but in large part the role of insects as vectors in the epidemiology of ASF in Europe is unclear and under debate (13–15).

The ASFV in highly resistant to environmental factors (such as pH) and can last over a year in blood, a few months in boneless meat and even a few years in frozen carcasses (1). Experiments confirmed excretion of ASFV in nasal, rectal and oral fluids as well as faces and urine of infected animals, thus causing contamination of feed, water, soil and environment (16). Contaminated pig feed was responsible for at least two independent introductions of ASFV in China in 2018 (17). Furthermore, ASFV is not sensitive to the process of decomposition of carcasses and survives up to 112 days in forest soil (1). In a study from Germany, soil pH, soil structure, and ambient temperature played a role in the stability of infectious ASFV. Infectious ASFV was demonstrated in specimens originating from sterile sand for at least three weeks, from beach sand for up to two weeks, from yard soil for one week, and from swamp soil for three days while ASFV was not recovered from two acidic forest soils (18). In Romania, contamination of water from Danube river is a suspected cause of ASF outbreak in a large farm with high biosecurity measures (19). Possibilities for infection of swine via ingestion of contaminated feed and water have been assessed, showing that liquid diet enhance ASFV infection (20).

For a better understanding and exploration of more effective prophylactic and reactive management actions against ASFV in Serbia, an individual-based approach is recommended to comprehensively investigate and consider all the local patterns of the disease, including characteristic outbreaks in wild boars, potential amplifying spots, virus sources, and other potential drivers such as habits of the human population, and vectors. The implementation of measures based on the knowledge is urgently needed (21).

In Serbia, it is shown that outbreaks and ASFV eradication processes in domestic pigs in poor, low-biosecurity are not dependent on ASF occurrence in wild boar whose involvement in ASFV transmission needs further investigation (3). Additionally, during the outbreaks in Serbia, observations revealed instances where ASF spread rapidly under epidemiologically unclear circumstances, despite the implementation of timely and comprehensive epidemiological investigations and measures. It was suspected that potential alternative pathways or drivers, facilitating disease transmission between locations, might be present including the role of environmental contamination and/or arthropod vectors. The role of these two potential ASFV transmission paths in Serbia is unclear and understudied. Hence, our study aimed to give further insight into transmission pathways of ASFV in Serbia through ASFV DNA detection in an environment and insects- flies during an outbreak. Furthermore, the role of adult flies (*Lucilia sericata* (*Diptera: Calliphoridae*)*, S. calcitrans*) in the mechanical transmission of ASFV was experimentally assessed in order to determinate a potential timeframe in real-time setting and laboratory conditions.

## Materials and methods

### Study area and description of sampling localities

**Figure 1:**
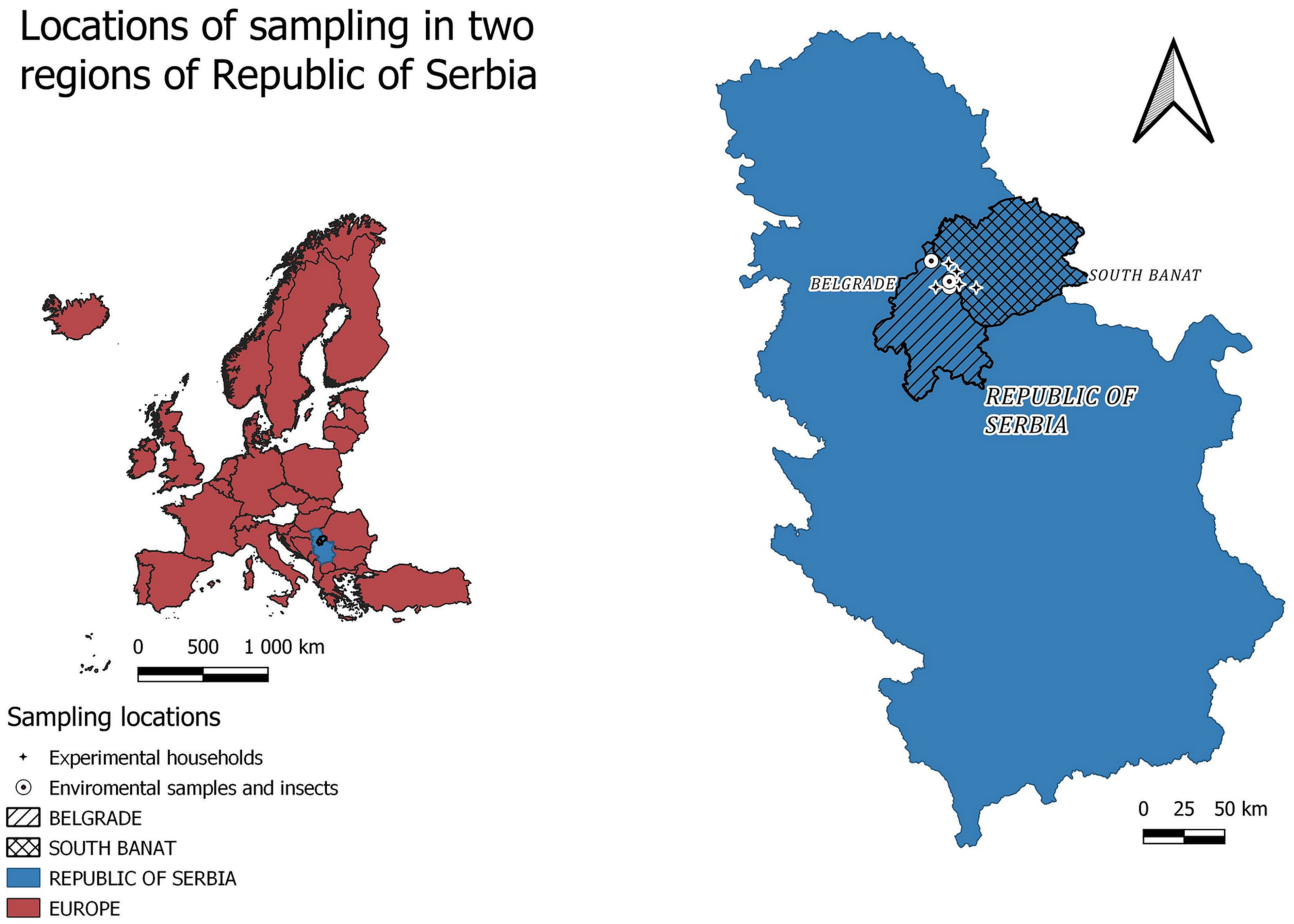
Locations of sampling in two regions of Serbia. Figure was produced using QGIS Version 3.28.3 Firence [QGIS.org (2024). QGIS Geographic Information System. Open Source Geospatial Foundation Project, “http://qgis.org”].

This study was performed in backyard pig production units and commercial farms consistent with aforementioned characteristics in two neighboring districts in Serbia (Belgrade city area and Southern Banat district) (Figure 1). All samples were collected during July and August 2023 in backyard pig production units (which in our study consisted of up to 5 pigs per unit), where ASF was previously detected by the National Reference Laboratory (NRL) at the Scientific Institute of Veterinary Medicine of Serbia (up to 48 h) before implementation of culling and disinfection measures (Figure 1). Established morbidity rate in households was between 50 and 70% depending on timepoint between the onset of the disease and the day of sampling.

The environmental samples (soil, feed, water, and environmental swabs from the pig barns) were taken from four localities, two in Belgrade city area (Duboka bara-Palilula 44.875905, 20.612613 and Borča- Put za Crvenku 44.861237, 20.438505) and two in Southern Banat district (Jabuka 44.943056, 20.593056 and Glogonj 44.983333, 20.533333). The soil samples (*n* = 6) were taken from three backyard pig production units, in the radius of 10 meters from barn where the affected animals stayed, based on the hypothesis of active contamination due to sewage leakage. From one household in the Borca locality, feed samples (*n* = 3) were taken from full feeders where a traces of commercial feed, grains and swill existed. Drinking water (*n* = 3) was sampled at two backyard pig production units in Glogonj and one in Jabuka village. Environmental swabs were taken directly at the pig pen with outdoor access (*n* = 12), and 30 m from the place where diseased animals lived (*n* = 3) in all four localities. Insect samples-pools up to 20 flies (*n* = 9) were taken at two localities (Glogonj and Duboka bara-Palilula), while pools up to 100 flies were sampled at a commercial pig farm, where ASF was confirmed (44.858669, 20.736289) directly near the entrance to the pig production objects (*n* = 3) and up to 30 m away (*n* = 3). Additionally, the *Muscidae* eggs (*n* = 2) and larval stages (*n* = 5) were collected from the carcass of swine improperly disposed by the road in Borča-Put za Crvenku.

The households hosting sticky fly traps and open Petri dishes containing minced meat mixed with humid cotton wads to prevent desiccation at high summer temperatures were offered at three locations in Belgrade area namely Reva (44.861237, 20.538505), Ovča (44.890923, 20.538998) and Besni fok (44.998121, 20.405613) during five days of field experiment in August 2023. These localities were chosen based on the intensive ASFV activity nearby and accessibility with consent of the owners.

Larvae of the L2 and L3 instar for ASFV transmission experiments were sampled at two backyard pig production units (45.015525, 20.385289; 44.861237, 20.438505), in Besni fok, Belgrade location.

Average summer temperature for wider Belgrade area in July 2023 was 24.7°C, while maximal temperatures during the peak of the day were up to 36°C based on the data of Republic Hydrometeorological Service of Serbia.

### Sample collection and analysis

Environmental samples of soil were collected by direct sampling of a superficial layer (up to 5 cm in depth) with a spatula in a quantity of 50 g to the plastic containers from the location near the stables where ASFV-infected animals were bred. The soil samples were thoroughly mixed in 10% suspension in a minimal essential medium (MEM, Gibco, ThermoFisher Scientific, Waltham, MA, USA) enriched with antibiotics and antimycotics (MycoZap, Lonza Bioscience, Basel, Switzerland) and centrifuged at 2000 × g for 30 min. Supernatant was decanted and used for further analysis.

Feed was probed from a feeder in 500 g quantity. In the laboratory, the feed was thoroughly mixed, then from each mixed sample, one gram of feed was further mixed in 10% suspension in MEM (MEM, Gibco, ThermoFisher Scientific, Waltham, MA, USA) with antibiotics and antimycotics (MycoZap, Lonza Bioscience, Basel, Switzerland), centrifuged at 2000 x g for 30 min, decanted, and frozen (at −80°C) until further use.

Drinking water was taken from the drinking troughs, from which the pigs drank, in 500 mL sterile containment. Water samples were collected from three locations, and brought to the Institute. The water samples were centrifuged at 5000 × g for 10 min, decanted, and frozen (at −80°C) until further use.

Equipment and metal surfaces, in the direct environment of ASFV-infected animals in pig barns, were swabbed using pre-wetted swabs with 0.9% natrium-chloride solution Swabs were submerged in 1 mL of phosphate-buffered saline (PBS) and subjected to homogenization. Subsequently, the samples underwent centrifugation at 1500 × g for 10 min. The resulting supernatants were carefully decanted and preserved at −80°C for subsequent analysis, following the protocol by Gallardo et al. (22).

Adult flies were collected manually using entomological nets, while on a farm sticky traps were set for the collection purpose. The flies were transferred to the NRL at the Scientific Institute of Veterinary Medicine of Serbia in cold chain. Representative subsample (*n* = 5 flies per sample selected by an entomologist) was taken for morphological insect identification. After morphological identification, the flies were pooled up to 100 flies based on observed morphological characteristics and stored at −80°C for subsequent analysis. *Muscidae* eggs and larval stages were collected using entomological tweezers, brought to the NRL, subsampled for confirmation of the fly species and stored at −80°C for subsequent analysis. Homogenization was performed using Tissuelyser II (Qiagen, Hilden Germany) at 30 Hz for 1 min.

### ASFV transmission trials in households which encountered ASFV outbreak

The attractant for flies for transmission trials in households which encountered ASFV outbreak consisted of Petri dishes containing minced beef meat (for human consumption, bought on the day when the traps were set), wads and water. Samples were left for three days exposed to contact with flying insects. Afterwards, Petri dishes were collected and transferred to the laboratory in hand freezers at +4°C. These samples were divided in three 1 g samples, homogenized in Tissuelyser II (Qiagen, Hilden Germany) at 30 Hz for 1 min.

### Preparation of the first-generation flies for ASFV transmission experiments

**Figure 2:**
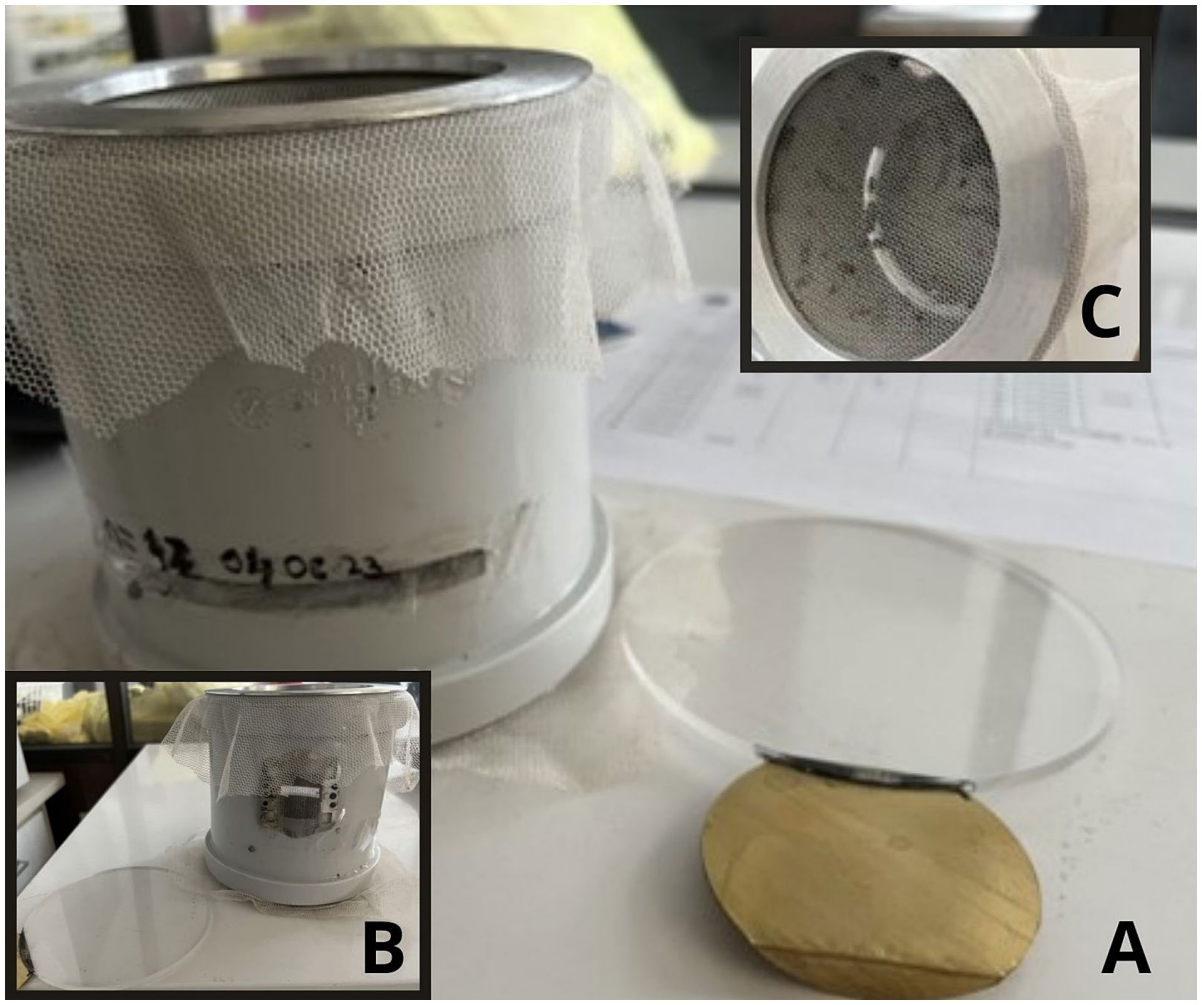
ASFV transmission experimental set-up: modified infection traps for adult flies – (A) front, (B) back, (C) above perspective. Figure was produced using Canva (2024), https://www.canva.com/design/DAGLIXnv1U4/EL8tjI6kDIVMqHwdWryjrQ/edit.

Larval instars L2 and L3 were taken from the carcasses of found dead pigs which tested positive for ASF. The collected larvae for the Besni fok location were split into two groups, one was tested for ASF, and the other was collected for rearing at the Insitute of Veterinary Medicine of Serbia. Larvae were selected based on the instars and morphological characteristics and placed in double containment. Larvae, pupae and adults were kept at 24–26°C, 60–70% relative humidity (RH). Larvae were fed with feed composed of 50% humid paper wads, and 50% minced cattle meat replaced every second day, while adults were offered 5% glucose solution *ad libitum*. Larvae L3 (*n* = 3) were tested for the presence of ASFV DNA using qPCR (23). A total of 42 pupae in the first experiment and 21 pupae in the second experiment were transferred into modified trial cages- with slight modifications to described in [(24); Figure 2]. Prior to ASFV transmission trials, adult flies (*n* = 3 per trial) were taken for morphological identification and molecular confirmation of species.

### ASFV transmission experiments

ASFV transmission trials were done in two separate consecutive trials. Three-day-old adult flies were deprived of glucose solution 24 h prior to experiments. They were offered a pork meat meal (1 cm^3^) spiked with 100 μL ASFV titre 6log_10_HAD_50_/mL. Before and after feeding, control samples of viral stock were taken and controlled for ASFV titre using qPCR quantification. The meat meal was kept for 24 h, then replaced on a daily basis with non-ASFV spiked meat to mimic transmission to non-infected animals up to 5 days after infection, depending on the available number of flies. Flies (*n* = 3) were taken for morphological insect identification to confirm identity. Day-0 samples (*n* = 3) for control purposes were taken after 1 h exposure to ASFV spiked meat.

### Identification of insect species

Morphological identification of third instar larvae and adult flies was performed using morphological identification keys (25–27).

For confirmation of morphological identification, a representative number of samples was analyzed using molecular biology methods. Flies were kept in 70% ethanol which was withdrawn prior to DNA extraction and insects were left in open 2.0 mL tubes to dry out, and homogenized in Tissuelyser II (Qiagen, Hilden Germany) at 30 Hz for 1 min. DNA extraction was performed using a Tissue DNA purification kit, EURX, Poland following the manufacturer’s instructions. Isolated DNA was kept at −20\u00B0C. Molecular identification of flies was performed using the protocol described in Folmer et al. (1994), which established “universal” DNA primers to amplify a fragment of the mitochondrial cytochrome c oxidase subunit I gene (COI) of invertebrates from 11 phyla.

Sanger sequencing was performed using the commercial service of the Macrogen company (Amsterdam, The Netherlands) and the Faculty of Biology, University of Belgrade (Belgrade, Serbia).

### Isolation of ASFV DNA from environmental samples and qPCR for ASFV detection in environmental samples and insects

Viral DNA extraction was performed utilizing the IndiSpin Pathogen Kit (Indical Bioscience GmbH, Leipzig, Germany), adhering strictly to the protocols specified by the manufacturer. To identify the ASFV genome, the study employed the primer and probe set delineated in the work by King et al. (23). The quantitative PCR (qPCR) reaction was formulated using 12.5 μL of Luna Universal Probe qPCR Master Mix (New England BioLabs, Ipswich, MA, USA), 1 μL of each primer at a concentration of 10 mM, 0.5 μL of a 10 mM probe, and 5 μL of the extracted DNA template. Nuclease-free water was added to adjust the total volume of the reaction mixture to 25 μL. The qPCR thermocycling conditions commenced with an initial denaturation at 95°C for 1 min, followed by a series of 40 cycles, each consisting of a denaturation phase at 95°C for 15 s and an annealing/extension phase at 60°C for 30 s.

## Results

### Morphological identification of flies and molecular confirmation of fly species

At two localities (Glogonj and Duboka bara-Palilula), subsamples of non-biting flies caught using entomological nets were morphologically identified as *L.sericata, Lucilia silvarum* (Diptera: Calliphoridae), and *Chrysomya albiceps* (Diptera: Calliphoridae) while flies in pools up to 100 that were sampled at the near of Stari Tamiš pig farm were identified as *M. domestica* and *S. calcitrans*. The molecular insect identification confirmed the presence of *L. sericata, C. albiceps* and *S. calcitrans*. Additionally, the collected muscidae eggs were identified as *C. albiceps and L. sericata,* while larval stages collected from the carcass of swine in Borča-Put za Crvenku were identified as *M. domestica* and *L. caesar* (Diptera: Calliphoridae) using molecular biology methods.

The adult flies taken for morphological identification prior to two transmission experiments were identified as *L. sericata*. After molecular insect species identification, a presence of *S. calcitrans* was confirmed in one sample.

### Detection of ASFV DNA

The environmental samples (soil, water, feed and swabs) were all tested negative for the presence of ASFV DNA.

The pool of Muscidae eggs (*C. albiceps*), and a pool of L1 instar larvae (*L. sericata*) collected at the carcass of diseased swine at the location Borča-Put za Crvenku tested positive for the presence of ASF DNA (Ct = 29.3 and 33.4).

All flies taken for control testing prior to experiments were tested negative.

All Petri dishes containing meat at three locations were tested negative for the presence of ASFV DNA.

One *S. calcitrans* fly sampled on day 1 post-infection tested positive (Ct-34.2) in the first experimental trial group.

## Discussion

Initial outbreaks of ASF in Serbia were detected in the domestic pig population (2). The main transmission paths in backyard pig production units seemed to be mostly independent of contact with wild boar population and related to human activities and unclear factors. Nevertheless by 2023 ASFV became present in eastern part of Serbia and had tendency of spreading westwards.

The data from the field suggest that adult flies have a role in the mechanical transmission of ASFV, but the knowledge of transmission dynamics or exact fly species involved is insufficient A recent EFSA entomological study in Romania identified 62.96% of *Stomoxys calcitrans* (*Diptera: Muscidae*) and 42.02% collected hematophagous biting midges (*Diptera: Ceratopogonidae*) pools as positive for ASFV DNA presence emphasizing the need of vector competence and virus transmission studies in different insect species (14). In Estonia, on a ASFV infected farm, viral DNA was detected in the housefly *M. domestica (Diptera: Muscidae)* and one *Drosophila* spp. (*Diptera: Drosophilidae*) (13). In another study *M. domestica*, *Calliphoridae (Diptera: Calliphoridae)* and stable fly *S. calcitrans (Diptera: Muscidae)* also tested positive for ASFV DNA presence (28). Mechanical transmission of ASFV by *S. calcitrans* has been experimentally shown to transmit ASFV to domestic pigs 1 and 24 h after feeding on infected material (29). However, in the same experimental work, transmission failed if the interval between feeding on infected material and uninfected pigs increased. Stable flies were also shown under experimental conditions to be able to transmit ASFV if ingested by naive domestic pigs (30). ASFV has been detected in various body parts of *S. calcitrans* for up to three days following experimental infection (31). Furthermore, the role of the common green bottle fly, *L. sericata (Diptera: Calliphoridae),* and the blue bottle fly, *C. vicina (Diptera: Calliphoridae), w*as experimentally shown when larvae experimentally bred on ASFV-infected spleen tissue. It was concluded that immature blowfly stages do not play a relevant role as reservoirs or mechanical vectors of ASFV unlikely adults (32). Recent study demonstrated dynamics of ASFV transmission in mosquitoes as representative of small blood feeding insects, tabanids and *S. calcitrans* at three different incubation temperatures (10°C, 20°C, 30°C) (33). It was proven that ASFV is viable in *S. calcitrans* at 48 h post infection sampling point at the temperature of 30°C, but not later (33), which is similiar to summer temperatures in Serbia. Since our result showed presence of ASFV DNA in *S. calcitrans* only at 24 h post infection at the temperature of 27 ± 1°C, we hypothesize that this result is in agreement with results of (33) and that the higher the temperature, the less viable ASFV is when transmitted via *S.calcitrans*. Nevertheless, our result comes from infection trial, and not from the field-collected insects and therefore further investigation is needed in the field conditions.

In our study, we were able to confirm ASFV DNA in field-collected eggs and larvae from carcasses of swine tested positive for ASF which is in correlation with results of (32). Subsequent sequencing proved these positive samples to originate from *C. albiceps* and *L. sericata* flies, respectively. These results are in correlation with Balmoş et al. (12) where among different fly species which were tested positive, most classified as belonging to *Calliphoridae* family. The adult flies are known to be able to mechanically spread ASFV in areas of up to 2–3 km between pig farms (28), and therefore might have an influence on ASFV transmission. However, in our study the samples (meat traps) from nearby households which encountered ASFV outbreak tested negative for ASFV DNA, highlighting the complexity of environmental transmission of ASFV. Nevertheless, ASFV transmission via flies as mechanical vectors could not be ruled out based on these results, since environmental conditions (e.g., high summer temperatures, placement of samples in yards) might have had influence on them results.

The role of *L. sericata* and *C. vicina* flies as mechanical vectors has been previously proven starting on larval feeding on ASFV infected tissue (32). In our approach, after the initial results of ASFV DNA positivity in field-collected *L. sericata* larvae, we used negative larvae morphologically identified as *L. sericata* to develop adult flies to be exposed to the known titre of ASFV with a goal to get insight into transmission dynamics in adult flies, i.e., duration of ASFV positivity which could imply the role in local ASFV transmissions observed in the field. Our results showed that no *L. sericata* flies tested positive for ASFV DNA, while the only fly identified as *S. calcitrans* by molecular insect identification was positive only on day 1 of the transmission experiment 24 h after the exposure to the ASFV. No further positivity was observed in both experiments. This result highlights again the potential role of *S. calcitrans* as a mechanical vector of ASFV. Nevertheless, the limitations of this study are that we were not able to prove if the ASFV was located on the fly or ingested, since no dissections were done, nor if the ASFV was live or not, while only molecular biology methods were used in viral detection. Therefore we could not establish whether ASFV transmission would be possible through ingestion of whole insect or by piercing the susceptible animal.

In our study, we were not able to detect ASFV DNA in environmental samples of soil, water, feed and swabs taken directly where the diseased animals were present. This might be due to the limited number of samples in this study [soil samples (*n* = 6), feed samples (*n* = 3), environmental swabs (*n* = 15)], but also might be due to the low ASFV contamination in the environment where sampling took place. It was shown that the applied study methodology is a critical issue when conducting stability testing (33). Furthermore, the time frame of sampling (from 48 to 96 h from the onset of clinical signs- during the culling procedure) might have influenced the outcome of this study since high viral loads in blood of ASFV infected animals start to be present from day five post infection (34, 35). Finally, sampling was performed during period of high summer temperatures in Serbia reaching 40°C, which might have also influenced the outcome, since it is shown that ASFV persistence is declining with the rise of temperature in feces and urine of ASFV infected pigs (36). The negative results obtained in this study do not exclude the possibility of ASFV transmission via contaminated surfaces or fomites. Olsen et al. found that naive pigs were not infected, if they were put in the pens where ASF-pos pigs had been staying 3, 5, or 7 days earlier, while naive pigs did get infected if inserted 24-h after removal of ASF-pos pigs. These pens were not cleaned and disinfected, while visible blood was removed in between (37). Environmental samples are effectively used for detection of ASFV in wild boars (38).

Effective management of ASFV outbreaks requires a comprehensive approach considering local disease patterns, wild boar outbreaks, potential amplifying spots, and human activities. In Serbia, low-biosecurity settings in rural areas with backyard production have been particularly challenging (39). In Serbia dominant transmission route in domestic pigs seems to be direct contact between infected and susceptible swine, together with human activity (40).

## Conclusion

ASF remains a significant threat to swine populations, with its persistence in the environment and potential vector-mediated transmission complicating control efforts. Continuous monitoring, genetic characterization of virus strains, and comprehensive management strategies are essential for effective ASF control. Understanding the roles of various vectors and environmental factors will be crucial in developing more targeted and effective interventions to prevent and mitigate ASF outbreaks.

## Data Availability

The raw data supporting the conclusions of this article will be made available by the authors, without undue reservation.
